# How 2D Nanoflakes Improve Transport in Mixed Matrix Membranes: Insights from a Simple Lattice Model and Dynamic Mean Field Theory

**DOI:** 10.1021/acsami.4c00661

**Published:** 2024-02-03

**Authors:** Tianmu Yuan, Lev Sarkisov

**Affiliations:** Department of Chemical Engineering, Engineering Building A, The University of Manchester, Manchester M13 9PL, U.K.

**Keywords:** gas separation, carbon capture, mixed matrix membrane, graphene, lattice model, statistical mechanics

## Abstract

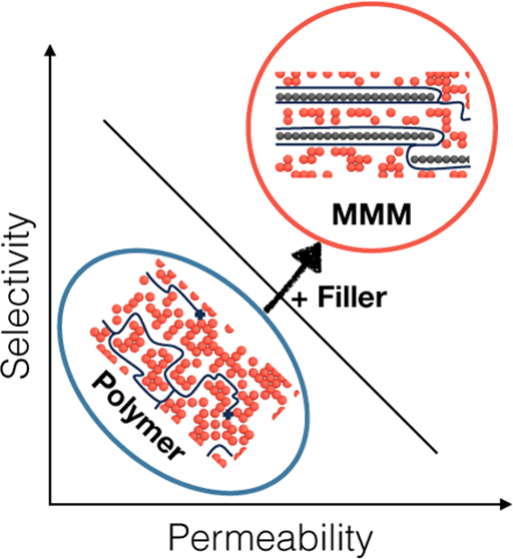

Mixed matrix membranes (MMMs), incorporating graphene and graphene oxide structural fragments, have emerged as promising materials for challenging gas separation processes. What remains unclear is the actual molecular mechanism responsible for the enhanced permeability and perm-selectivity of these materials. With the fully atomistic models still unable to handle the required time and length scales, here, we employ a simple qualitative model based on the lattice representation of the physical system and dynamic mean field theory. We demonstrate that the performance enhancement results from the flux-regularization impact of the 2D nanoflakes and that this effect sensitively depends on the orientation of the nanoflakes and the properties of the interface between the nanoflakes and the polymer.

## Introduction

The mission of this article is to develop a simple theoretical model which would elucidate the molecular mechanism by which adding nonporous structural elements such as 2D graphene and graphene oxide (GO) nanoflakes to the polymer improves gas separation performance of these materials beyond the formidable Robeson bound.^1,2^

Mixed matrix membranes (MMMs) emerged in the 1970s as promising materials for gas separations.^3^ The basic idea of an MMM is to combine low cost, mechanical robustness, and high processability of polymers with excellent sorp-selectivity and permeability of porous crystalline materials such as zeolites and, later, metal–organic frameworks (MOFs). Indeed, these novel materials showed promise to overcome the performance limitations of the conventional polymers typically delineated by the Robson bound for various gas pairs. In this scenario, the polymer assumes the role of structural support, while separation predominantly occurs via the pathways formed within the filler phase. However, a constraint exists concerning the filler loading as the membrane must retain its mechanical integrity to be viable for incorporation into an industrial membrane module. A recent study demonstrated the feasibility of producing hollow fiber membrane modules comprising MMMs possessing a filler volume fraction exceeding 80%, where MOFs are employed as the filler material.^4^ This advancement bears significant ramifications for membrane technology, as it offers the prospect of fabricating membranes predominantly composed of filler materials. Nevertheless, the generality of this approach and whether it can be extended to other fillers and polymers still need to be assessed.^4^

Extensive further experimentations with polymer–filler pairs led to a discovery that filler elements that do not possess their own porosity, such as 2D graphene and GO nanoflakes, may also enhance the separation performance of the hybrid material.^5−36^

**Figure 1:**
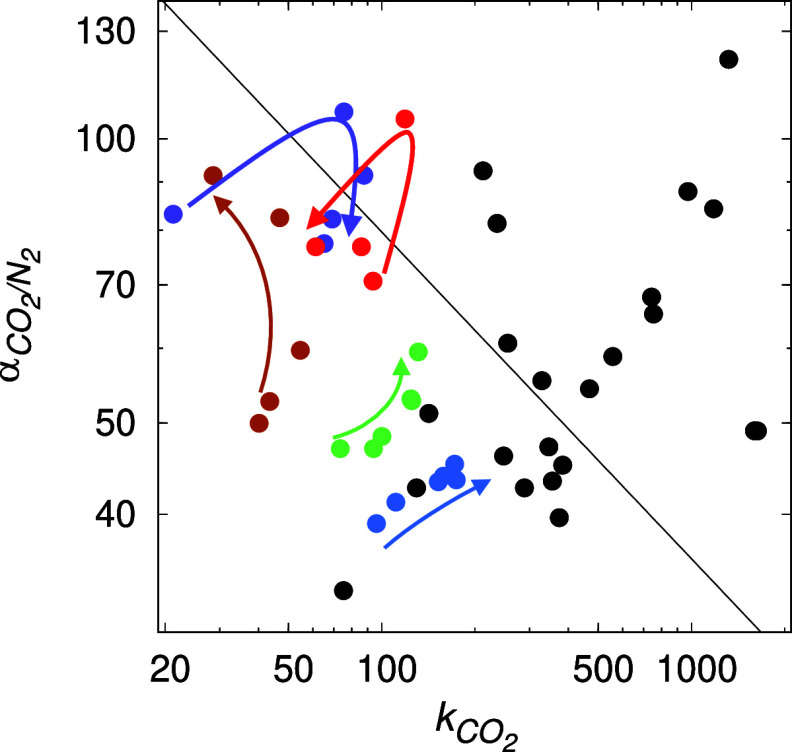
Robeson plot for the CO_2_/N_2_ gas pair. Here,  is the perm-selectivity for CO_2_/N_2_ and *k*_CO_2__ is the permeability for CO_2_. Each symbol represents an experimental MMM with 2D fillers such as graphene or GO.^5,6,9,13,14,19−21^ The black line is the 2008 Robeson upper bound.^2^ The green,^6^ purple,^5^ red,^13^ brown,^9^ and blue^19^ symbols are complemented with the arrows of the same color to highlight the change in the separation performance as a function of the increasing filler loading. See the main text for more details.

This is illustrated in Figure 1. Indeed, the integration of 2D materials with polymers leads to changes in the separation characteristics of the resulting membranes. The colored arrows in Figure 1 highlight the performance variation in selected systems as a function of increasing filler loading. In particular, Li et al. (represented by the green symbols in Figure 1), used GO to modify poly(ether-block-amide) (Pebax). The resulting modified membrane exhibited increasing permeability and perm-selectivity as the concentration of the filler increased.^6^ A comparable trend of similar systems was also observed by Zhang et al. (depicted by the blue symbols in Figure 1).^19^ In another example, Shen et al., used GO to modify polyvinyl amine/chitosan (represented by the purple symbols).^5^ As the concentration of the filler increased, their MMMs crossed the Robeson boundary; however, further increase in the amount of filler led to a deteriorating performance.^5^ Similar observations were noted in the study by Dong et al. using GO and Pebax (indicated by the red symbols).^13^ At the same time, it is worth noting that many experimental systems exhibit scattered patterns, and it becomes impossible to establish a clear trend regarding their performance as a function of filler concentration.^12,13,19,21,25^ This summary leads to the fundamental questions: how do 2D materials contribute to the enhancement of separation performance in MMMs? What is the molecular origin of this contribution?

Numerous research endeavors have been undertaken to elucidate the role of fillers in MMMs.^37−39^ Broadly, these investigations can be categorized into three primary research domains.

The first category centers around macroscopic models, such as Maxwell^40−42^ and Bruggeman,^43^ and various derivatives thereof, employed to predict the separation performance of heterogeneous membranes.^37,38^ These theories are based on the premise that fluid transport in heterogeneous materials can be treated analogously to the thermal and electrical conductivities in a composite medium. Despite the widespread application of the original Maxwell and Bruggeman theories to predict transport in MMMs due to their simplicity, their accuracy is limited. This arises from several assumptions in these theories such as the uniform distribution of identical spherical filler elements, which do not accurately capture the complexity and heterogeneity of real MMMs. More sophisticated theories have been developed to rectify these limitations to take into account the distribution, shape, and orientation of the filler particles.^37,38^ Some of the more advanced theories also attempted to include the interface between the filler elements and the polymer phase into consideration. In particular, one proposed strategy is to represent interfaces as an independent phase in addition to the filler and polymer phases.^37^ In the resulting models, the overall transport properties are conceptualized as the behavior of a three-phase system, applying principles similar to the two-phase models.^44−46^ While these approaches incorporate interfacial effects in MMMs and have demonstrated success in predicting permeabilities accurately, they necessitate prior knowledge of transport properties in the interfacial region, which are often challenging to obtain.

The second category involves employing computational fluid dynamics (CFD) approaches, utilizing numerical modeling to explicitly depict fluid transport patterns within heterogeneous materials and predict membrane performance.^47−57^ This approach enables the consideration of a fully defined MMM morphology, offering efficiency in constructing a comprehensive set of systems with varying conditions, shapes and sizes of fillers, filler loading, and distributions. For instance, several studies investigated the influence of the orientation of both nonporous^47^ and porous fillers^58,59^ on the fluid transport properties inside a heterogeneous membrane. A common practice in the construction of CFD models is to ignore specific effects and properties of the interface between the filler elements and the polymer phase. Yet, this region of the system may have very peculiar features affecting the overall performance of the composite MMM.^60^ For example, pore-blocking is one of the commonly stipulated effects at the interface between the micro- or mesoporous filler element and polymer phase. Pore-blocking results in fluid transport pathways obstructed in both phases and hence diminished permeability of the MMM.

Molecular models of transport in MMMs comprise the final category. Here, the polymer and filler materials, and the interface between them are represented with atomistic resolution.^17,28,30,61−74^ This approach offers a molecularly detailed picture of the adsorption and diffusion processes in the interfacial region. Interestingly, it may challenge our perception of interfacial regions as necessarily detrimental defects. For example, a notable study by Fan et al. investigates the accumulation of CO_2_ at the interface between PIM-1 and NUS-8, leading to high CO_2_/N_2_ and CO_2_/CH_4_ selectivity.^75^ This suggests that certain defects might actually be beneficial for enhancing transport in MMMs, sparking interest in systematic approaches to engineering defects and interfacial regions with specific characteristics.

The majority of previous studies on fluid separation in MMMs using molecular models have focused on equilibrium conditions. In these studies, membrane solubility (*S*) is typically determined using grand canonical Monte Carlo simulations, and diffusivity (*D*) is obtained from the equilibrium molecular dynamics. Permeability (*k*) is then derived based on the solution-diffusion model, i.e., *k* = *DS*. This approach, while informative, does not capture the realistic membrane process, wherein a density/pressure/chemical potential gradient exists across the membrane, leading to a nonequilibrium steady-state condition. To address this limitation, several nonequilibrium molecular dynamics (NEMD) methods have been developed to study transport in MMMs and the role of interfaces in heterogeneous materials. For instance, Yazaydin and co-workers conducted NEMD simulations on CH_4_/H_2_ separation using an MMM constructed from slabs of polymer (PIM-1) and filler (ZIF-8) materials.^68^ Similarly, Kong and Liu investigated the separation of CO_2_/N_2_ using a combination of porous organic cages and PIM-1.^66^ While these studies have significantly enhanced our understanding of fluid transport in heterogeneous media, they are computationally demanding. Given current computational limitations, conducting a comprehensive analysis of a diverse set of structures with different filler loadings, geometries, and distribution arrangements remains prohibitive. This constraint hinders the decoupling and understanding of the factors responsible for transport phenomena in MMMs.

To summarize, this review highlights the gap in the spectrum of current models available for studies of MMMs. On one hand, the macroscopic and CFD models allow for an efficient exploration of the impact of size, volume fraction, geometry, and orientation of filler elements on the performance of MMMs, but tend to oversimplify molecular processes at the interface. In contrast to that, molecular models reveal important processes at the interface but are computationally expensive to explore nonequilibrium transport across the whole membrane.

To answer the questions posed in this study, we require a model that provides sufficient details and control of the processes at the interface between the phases in MMMs and at the same time is computationally efficient to explore nonequilibrium transport across a diverse set of systems, ranging in concentration and orientation of nanoflakes.

In this work, we introduce a lattice model combined with dynamic mean field theory (DMFT) to achieve this objective. Briefly, our model considers a system of sites arranged in a simple cubic lattice, where each site can be occupied either by a fluid particle or a solid particle representing the components of the MMMs, or remain empty. We only take into account the nearest neighbor interactions. The mean field treatment of the Hamiltonian of this lattice system considers an averaged fluid density on each available site instead of the explicit occupancy, and as a result, this leads to an analytical expression for the density distribution of the system at a particular temperature and chemical potential or bulk pressure of the fluid. The DMFT stems from the Kawasaki dynamics,^76−78^ where fluid transport is modeled via a hopping mechanism between neighboring sites, and the acceptance of each hop is based on the Metropolis criteria (see Methods section for more details). The DMFT allows us to explicitly consider transport across a system, driven by chemical potentials or pressure gradients. These nonequilibrium conditions imitate conditions across a membrane. This method has been applied extensively to study the adsorption/desorption hysteresis, capillary condensation, and diffusion in porous materials.^79−100^ Previously, we have demonstrated the utility of the DMFT in studies of single component fluid transport in simple slit pores,^101^ and complex, heterogeneous media.^60^ In a follow-up study, we investigated two-component systems to explain separation phenomena in sorption-driven membrane processes.^102^

## Methods

### Mean Field Theory

The theoretical framework applied in the work closely follows the work by Edison and Monson,^88,95^ and we refer the readers to the original articles for additional details.

#### Static Mean Field Theory

We start by considering a simple cubic lattice system with nearest neighbor interactions under an external field ψ. Each site can be occupied by either a fluid particle, a solid (representing either polymer or filler material) particle, or being empty. We only consider nearest neighbor interactions. Therefore, the Hamiltonian for a two-component system with fluid species *A* and *B* in an external field ψ can be written as
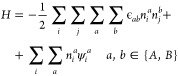
1where *a* and *b* denote the fluid species and can be either *A* and *B*, ϵ_*ab*_ describes the interaction between fluid *a* and *b*. The fluid occupancy for site *i* is denoted as *n*_*i*_ which can be either 0 (unoccupied) and 1 (occupied), and *j* represents the nearest neighbor of site *i* (*j* ≠ *i*). The external field at site *i*, ψ_*i*_ can be expressed as
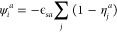
2where ϵ_*sa*_ describes the solid–fluid interaction between solid *s* and fluid *a*, and η_*j*_ is the solid occupancy at site *j*. The value for η can be either 0 (occupied) or 1 (unoccupied).

The mean field treatment^103^ leads to the following expression for the Hamiltonian
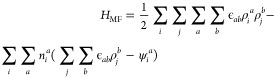
3where ρ = ⟨*n*⟩ is the dimensionless average occupancy or the density. The mean field grand potential of the system can then be written as
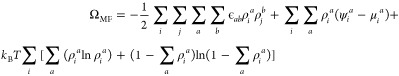
4where μ is the chemical potential and *k*_B_ is the Boltzmann constant. The minimization of the grand potential with respect to density gives the density distribution at equilibrium
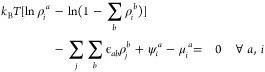
5

The equation described above can be solved iteratively for a density distribution at fixed chemical potentials for the individual species at a given temperature.

#### Dynamic Mean Field Theory

The dynamic behavior of the system closely follows the work by Gouyet et al.^104^ and Edison and Monson.^95^ The readers are referred to the original article for additional details.

We start by expressing the change in density at site *i* with respect to time
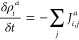
6where *J*_*i*,*j*_^*a*^ is the flux of species *a* from site *i* to its nearest neighbor *j*. Considering the Kawasaki dynamics which generates dynamics via nearest neighbor hopping processes, the flux from site *i* to its nearest neighbor *j* can be formulated as
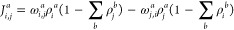
7where ω_*i*,*j*_^*a*^ is the transition probability of hopping for species *a* from site *i* to *j* based on the Metropolis criteria. This can be expressed as
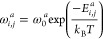
8where
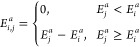
9and
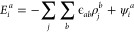
10

In the case of a steady-state condition , the flux can be rewritten as
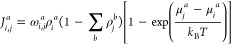
11

Both eqs 7 and 11 can be used to determine the dynamics transport behavior of the system. Throughout this work, unless otherwise specified, eq 7 will be used.

### Model Details

#### Interaction Parameters

From the chemical composition point of view, the system of interest includes two gas species, *A* and *B*, and two components of the MMMs: the polymer (*p*) and filler (*f*) components. Therefore, the complete set of parameters to describe interactions in the system should include fluid–fluid interactions for the two species (three parameters) and solid–fluid interactions for two gas species and two types of solid sites (four parameters). To make the process of setting the model tractable and systematic, we set the *A*–*A* interactions as our unit of energy, ϵ_*AA*_ = 1 and all other parameters of interactions are expressed in these units. In particular, starting from this point, the *B*–*B* interactions are scaled with respect to *A*–*A* to reflect the relative values of the bulk critical temperatures of CO_2_ and N_2_, ϵ_*BB*_ = 0.415, following our previous study.^102^ Now, let us consider the interaction between filler species *f* and fluid species *A* and *B*. Specifically, we adopt an interaction strength of ϵ_*fA*_ = 3 for species *A*, imitating interaction between CO_2_ and activated carbon surfaces.^101^ For species *B*, we set ϵ_*fB*_ = 1.93, scaled based on the critical temperature ratio of CO_2_ and N_2_, with .

A total of four combinations of ϵ_*pA*_ and ϵ_*pB*_ are created to reflect different scenarios of polymer–fluid interactions. In particular, *C*_1_ is the case where both fluids interact strongly with the polymer, *C*_2_ is the case where both fluids interact weakly with the polymer, and *C*_3_ is the case where species *A* interacts strongly with the polymer, and species *B* interacts weakly with the polymer. In comparison with filler–fluid interactions, the interactions between species *A* and the polymer sites are stronger in the *C*_1_ and *C*_3_ cases, but weaker in *C*_2_ case. Finally, we consider the fourth case, *C*_4_, where the ϵ_*fA*_ = ϵ_*pA*_ and ϵ_*fB*_ = ϵ_*pB*_. The last case allows us to focus specifically on the impact of the fillers on the structure of the flux in the system as, at the same total solid density, this will be the only factor differentiating the original polymer structure and the polymer structure modified with filler elements. The complete set of these parameters follows our previous study^102^ and is provided in Table 1.

**Table 1:** Parameters of Polymer–Fluid Interactions in *C*_1_–*C*_4_ Models

	*C*_1_	*C*_2_	*C*_3_	*C*_4_
ϵ_*pA*_	5.3	1.7	5.3	3
ϵ_*pB*_	3.413	1.095	0.623	1.93

#### System Configurations

**Figure 2:**
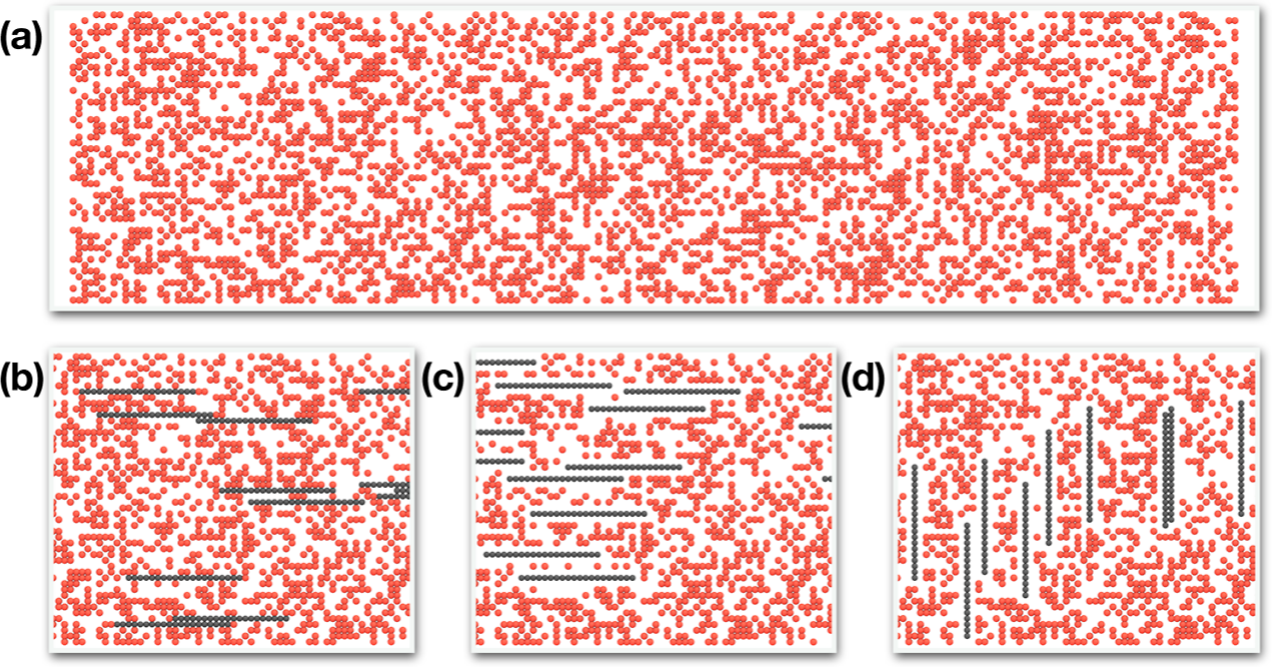
Computer visualizations of examples of lattice models of MMMs. In this figure, red spheres represent the polymer phase while gray spheres depict filler elements. In panel (a), a polymer with ϕ_*p*_ = 0.40 is illustrated. Panels (b–d) showcase MMMs derived from the polymer in panel (a). Panels (b) and (c) feature MMMs with filler elements in the horizontal orientation, exhibiting interfacial gaps (IGs) of 0 and 1σ, respectively. Panel (d) presents an MMM with filler elements in a vertical orientation (perpendicular to the total flux in the system) and IG = 1σ. See the main text for more details.

In the language of the lattice model, the polymer and filler materials are represented by a lattice site occupied by a solid particle of the specified type, either *p* or *f*, respectively. We constructed the continuous polymer phase by randomly distributing polymer particles on the lattice sites. An example is illustrated in Figure 2. Although not atomistically detailed, this setup captures some essential features of a glassy polymer: it is a rigid, amorphous structure, and the fluid pathway is disordered. The volume fraction of the polymer particles is represented by ϕ_*p*_. This is varied between 0.25, representing a relatively porous system, and 0.40, which corresponds to the percolation threshold of a cubic lattice system. Three independent configurations are created for each ϕ_*p*_ and all the properties reported are averaged over these configurations.

All system sizes are 200σ × 50σ in *x* and *z* dimensions, respectively, where σ is the unit lattice space. In the lattice model, each site can be occupied by 1 molecule. If we take the molecular diameter of CO_2_, *d*_CO_2__ ≈ 0.33 nm, then the systems above would roughly be 70 × 20 nm in real units. This choice of the system size is based on a series of preliminary studies of how the reported transport properties depend on the system size. We observed no changes in the properties of the systems exceeding 200σ × 50σ in their dimensions. In the *y*-axis, the system is one lattice site thick and is repeated in periodic boundary conditions. This leads to effective 2D systems. Here, we use this opportunity to emphasize that the presented model is not meant to be a coarse-grained version of the molecular structure of the actual membrane. The model is drastically oversimplified, it cannot quantitatively correctly capture properties such as the polymer porosity and fractional free volume. Nevertheless, it serves as an efficient and qualitative tool to investigate the fundamental aspects of fluid transport properties in complex, heterogeneous media. It also facilitates the exploration of the intricate interplay between polymer porosity, solid–fluid interactions, filler volume fraction, and distribution patterns.

In our lattice model, since the *y*-dimension of the system is one lattice site thick, repeated in periodic boundary conditions, the 2D nanoflakes are simply represented as a string of filler sites. In the current model, each string is 20σ long, which would be roughly 10 nm in real units. It is worth noting that actual graphene or GO nanoflakes typically range in size from hundreds of nanometers to micrometers.^6,9^ Although the systems under consideration are relatively small, given the fact that this is only a qualitative tool and we are not interested in the atomistic details, we argue that our chosen system size is sufficiently large to investigate fluid transport properties within heterogeneous media.

In Figure 2b–d, we show typical systems featuring filler elements, represented by strings of solid sites. Experimentally, the synthesis of MMMs is a delicate process associated with a number of technical challenges such as the formation of defects and nonuniform distribution of the filler component. In this study, we assume the MMMs are well-mixed (e.g., no agglomeration of fillers); therefore, a random distribution of the fillers is employed. The volume fraction (%) of the fillers, ϕ_*f*_, is varied between 0 and 30%, calculated as the number of sites occupied by filler particles, divided by the total number of lattice sites in the system. Further increase in the volume fraction of the fillers leads to model structures that resemble a collection of slit pore channels of various width, and this does not represent the physics of the systems we want to capture in this study.

We also ignore large defects such as air bubbles on the scale of micrometers, as it is beyond the scope of our model. Instead, we focus on the nature of the interface between the filler elements and the polymer. In particular, in the scenario of strong adhesion, one would expect little or no free volume to form at the interface. However, other adhesion scenarios imply possible gaps or voids between the fillers and the polymer structure.

To take these scenarios into account, we incorporate IGs of different sizes between the filler elements and polymer sites. In particular, an IG of 0, IG = 0, means that to insert the filler element at a particular location in the polymer, we only remove the polymer sites that occupy the location intended for the filler element. However, for IG = 1σ, and 2σ, we also remove all the polymer sites within a single layer and double layer around the filler element, respectively. Examples of systems with IG = 0 and 1σ are shown in Figure 2b,c, respectively. As a result of the introduction of the filler elements and IGs, some of the polymer sites are removed leading to the new fraction of polymer sites ϕ_*p*_^′^. The total fraction of solid phase in the composite MMM system is defined as ϕ_*s*_ = ϕ_*p*_^′^ + ϕ_*f*_ and this property is summarized in the Supporting Information (SI) for all systems under consideration.

The orientation of the fillers is also an important aspect that we explore. Due to the limitation of the cubic lattice model, only two orientations of the filler elements are possible: parallel and perpendicular to the fluid flow, providing two limiting scenarios and behaviors, and a more realistic picture would exhibit some combinations of these scenarios. Examples of these configurations are shown in Figure 2c (horizontal orientation),d (vertical orientation), respectively.

#### Nonequilibrium Steady State Properties

**Figure 3:**
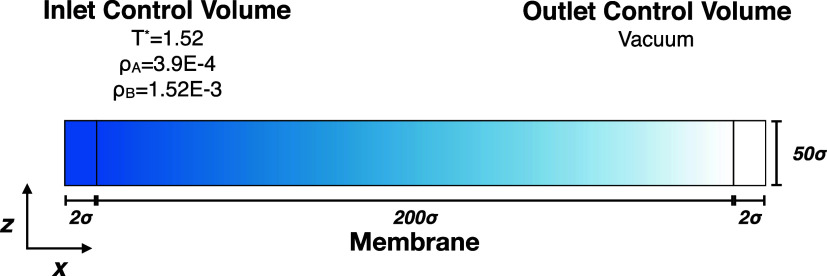
A schematic diagram of the nonequilibrium setup. The inlet and outlet control volumes are placed on the left and right-hand sides, respectively. The 200 × 50 σ^2^ membranes are placed in the middle. The control volumes are made of two layers of lattice sites.

The objective of this study is to explore how the presence of nonporous fillers influences the flux distribution, permeability, and perm-selectivity of a model membrane, compared to the unmodified reference system. To explore these properties under nonequilibrium steady-state conditions, we devise a setup that is the DMFT analog of the grand canonical molecular dynamics with dual control volumes. Specifically, our system consists of the model lattice membrane and two bulk lattice control volumes on the opposing sides of the membrane, as depicted in Figure 3. Although our theoretical framework remains qualitative, our objective was to draw parallels between real carbon capture conditions in power plant flue gas and the lattice gas phase diagram. Specifically, we configured the membrane inlet conditions to replicate the composition of exhaust from a coal power plant, featuring 15% carbon dioxide (component *A*) and 85% nitrogen (*B*) at 1 bar and 308.15 K. Considering the mean-field prediction for the critical temperature of a simple cubic lattice, denoted as *T*crit^*^ = 1.5, we scale the system’s temperature relative to the critical temperature to match the temperature ratio of flue gas to the critical temperature of carbon dioxide, resulting in . In the lattice model, the feed concentration translates to an inlet with ρ_A_^*^ = 3.9 × 10^–4^ and ρ_B_^*^ = 2.21 × 10^–3^. The chemical potentials for each component are μ_A_^*^ = −11.94 and μ_B_^*^ = −9.30, while the outlet condition assumes a vacuum with ρ_A_^*^ = ρ_B_^*^ = 0. We maintain a consistent time scale of ω_0_ = 0.2 for all species throughout our study. We follow our previous studies in which both fluid species have the same hopping rate. This allows us to investigate the separation phenomena resulting solely from the interaction patterns in model MMMs, decoupling them from the kinetic effects. With this framework, we explore the permeability and perm-selectivity as a function of solid–fluid interaction strength, filler loading, the nature of the interface between the nanoflakes and polymer, and the orientation of the nanoflakes.

The convention of the calculation closely follows our previous study.^60,101,102^ The flux from site *i* to its neighbors *j* of species *a* (where *a* can be either *A* or *B* in this work), *J*_*i*,*j*_^*a*^, is defined in eq 7. We note here that the net flux in both *y* and *z* directions is zero due to the absence of the chemical potential gradient along these axes. Hence, we can use the component of the flux in the direction of the fluid flow (+*x* direction), *J*_*a*,*i*_^+*x*^, and define the total net flux of the component *a*, *J*_*a*_, as the sum of the *J*_*a*,*i*_^+*x*^ fluxes along the *yz* plane
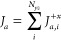
12where *N*_*yz*_ denotes the total number of sites in the *yz* plane. Using this definition of the flux, the permeability of species *a*, *k*_*a*_, is calculated in this work according to eq 13
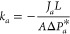
13where Δ*P*_a_^*^ is the pressure drop of component *a* across the membrane in dimensionless units, *L* is the length of the system, and *A* is the cross-sectional area. Since we have a 2D system, the cross-sectional area is simply the width of the system in the *z* direction. Since all properties in eq 13 are dimensionless, the permeability is also a dimensionless property in this study, reported throughout in arbitrary units. The perm-selectivity is, therefore, a ratio between the permeabilities
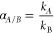
14To provide insights into the molecular origin of the enhanced performance of the nanoflake-modified membranes, we also visualized the fluid flux of the system. The local selectivity difference (Δα_*A*/*B*_) is also calculated and visualized to further aid our understanding of the separation phenomena. In short, the local selectivity α_*i*,*A*/*B*_ can be calculated at each site *i* as follows
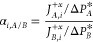
15Therefore, the local selectivity difference is defined as
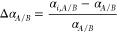
16

## Results and Discussion

In this study, we extend our investigation of the two-component gas systems to explore the role of 2D nanoflakes in separation phenomena in MMMs.

**Figure 4:**
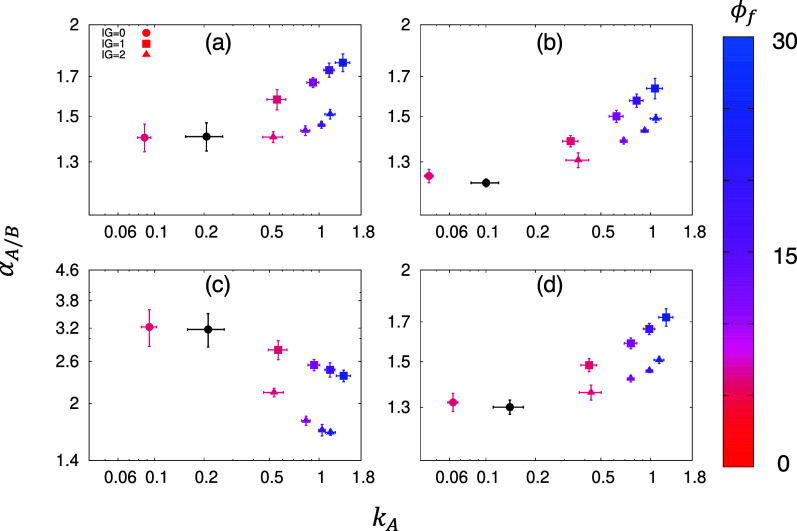
Perm-selectivity as a function of the permeability of *A*. Panel (a–d) shows the MMMs based on *C*_1_–*C*_4_ models of polymer–fluid interactions, respectively. The system with ϕ_*p*_ = 0.35 of polymer sites is taken as the reference membrane system. The black symbols indicate the properties of the reference, unmodified membrane. The circles, squares, and triangles correspond to MMMs with IG = 0, 1, and 2, respectively. The error bars are standard deviations and the color bar indicates the volume fraction (%) of the filler.

Figure 4 shows the perm-selectivity as a function of permeability for MMMs with horizontally oriented filler elements. The MMMs shown in this figure are based on a system of ϕ_*p*_ = 0.35. The black circles indicate the properties of the reference polymer membranes (ϕ_*f*_ = 0.0), averaged over three independent configurations. The circle, square, and triangle symbols correspond to MMMs with IG = 0, 1, and 2σ, respectively. The varying color of these symbols indicates the change in the volume fraction of filler elements, according to the side color bar. Each data point for MMM is averaged over 9 independent configurations with the exemption of nonpermeating membranes. The error bars are standard deviations.

Let us first comment on the reduced number of configurations sampled for MMMs with an IG = 0. We emphasize that the total number of configurations is still the same; however, we excluded from this analysis configurations that do not exhibit any flux due to the absence of percolation diffusion pathways. This is due to the fact that a system with zero flux cannot be located on the Robeson plot, as the perm-selectivity, , is undefined when *k*_*B*_ = 0. The nature of the diffusion pathways in a polymer is random and disordered, and adding a nonpermeable structural element to the system that features no space between the element and the polymer phase (perfect adhesion) significantly increases the chances of blocking the transport pathway. This agrees with the findings from our previous study.^60^ As for the configurations that remain percolated, the incorporation of the filler simply reduces the permeability, meaning that the original pathways are partially blocked.

**Figure 5:**
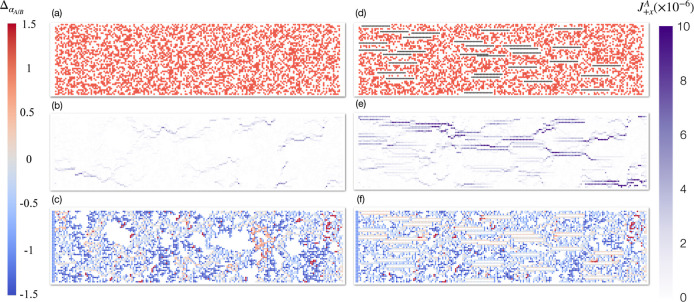
(a) Configuration snapshot of a reference polymer membrane (ϕ_*p*_ = 0.40), and visualization of its flux (b) and local selectivity difference (c) distribution. (d) Snapshot of an MMM based on the reference membrane with ϕ_*f*_ = 6% and IG = 1. Its flux and local selectivity difference distribution are demonstrated in (e,f), respectively. The color bar shows the local selectivity difference and flux in the +*x* direction in dimensionless units on the left and right sides, respectively.

The presence of IG dramatically changes the fluid transport in MMM structures. For all the cases we observed, given a reasonable filler volume fraction (ϕ_*f*_ ≤ 30%), higher loading of the filler ϕ_*f*_ increases the permeability of the MMMs; and in the cases other than *C*_3_, it also increases selectivity. To gain insights into the underlying mechanisms, we visualize the flux and local selectivity difference distributions in the membranes, shown in Figure 5. Panel (a) shows the snapshot of a pure polymer membrane with ϕ_*p*_ = 0.40 and ϕ_*f*_ = 0.0%, while panel (b) demonstrates its flux distribution of fluid *A* in the direction of fluid flow (+*x*), and panel (c) is the distribution of the local selectivity difference within the pure polymer membrane. Panel (d) demonstrates a typical MMM (ϕ_*f*_ = 6.0% and IG = 1) system based on the polymer from panel (a). The total solid fraction (polymer and filler combined) of this MMM is ϕ_*s*_ = 0.38 (see the SI for further details). The flux and local selectivity difference distributions for this system are presented in panels (e,f), respectively. By comparing panels (b,e), we can clearly observe that incorporating IGs with fillers creates well-defined pathways that enhance fluid transport by introducing smooth channels, reducing flow resistance, and minimizing free energy barriers associated with the polymer’s complex geometry. As a result, this leads to significantly enhanced fluid transport in MMMs. The visualization of the local selectivity difference further demonstrates that the selectivity is mainly concentrated in the fluid channels around the fillers, while the selectivity in the polymer membrane is dominated by random, narrow, and disordered pathways.

This observation poses an additional question: is the improved performance a result of the properties of the filler elements (in other words, specific filler–fluid interactions), the induced regularization of the flow, or simply an outcome of the reduced polymer phase volume fraction? To answer this question, we consider two additional systems. In the first system, we consider the *C*_4_ model of interactions. In this model, the filler–fluid interactions are exactly the same as polymer–fluid interactions for all fluid species. Furthermore, we ensure that the total solid fraction of polymer and filler sites (ϕ_*s*_) is approximately the same as the reference system, ϕ_*p*_ = 0.35.

Exploring the flux in this system decouples the structuring effect imposed by the filler elements from the possible influence of favorable filler–fluid interactions. The results are shown in Figure 4d where it contains results from MMMs with ϕ_*f*_ between 12 and 18%. As evidenced, for cases where the filler and polymer share the same interaction strength, the addition of fillers with IGs still increases the permeability and perm-selectivity.

In the second system, we start with the system described above and remove the filler elements completely, leaving “holes” in the structure in place of filler elements and leading to a system with a lower overall density. Considering this system tests whether the enhancement of the flow in the structure with fillers is a consequence of effectively higher free volume, produced by the introduction of the IGs, as we expect that in the system with “holes” this effect will be magnified even further. By comparing the separation performance of the polymer configurations with the filler elements removed with the MMMs, we found that both permeability and perm-selectivity of the MMMs (*k*_*A*_ = 1.05, α_*A*/*B*_ = 1.70) exceeds the polymer-with-holes system (*k*_*A*_ = 0.42, α_*A*/*B*_ = 1.08) and the reference polymer with ϕ_*p*_ = 0.35 (*k*_*A*_ = 0.12, α_*A*/*B*_ = 1.31). This suggests that the main impact of the filler elements is the regularization of the fluid flux by creating directional diffusion pathways.

The arrangement of the nanoflakes in the MMM is one important aspect, as it dramatically influences the separation performance. Experimentally, it was found that, for nanoflakes aligned parallel (horizontal in our study) to the fluid flow, the permeability is significantly increased.^34^ This observation is consistent with the results presented in Figure 4 in our study. Conversely, the same experimental work revealed that when the fillers are arranged perpendicularly to the fluid flow, it has minimal impact on the separation performance.^34^ Previously, our investigations indicated that inserting elongated filler elements in the orientation perpendicular to the fluid flow in the membranes has a detrimental effect on the fluid transport as it effectively obstructed the fluid pathway.^60^ However, we did not consider the effect of the IGs. In this study, we created cases where the filler elements are aligned vertically and also feature IGs of 1 or 2 lattice sites between the filler and polymer sites.

**Figure 6:**
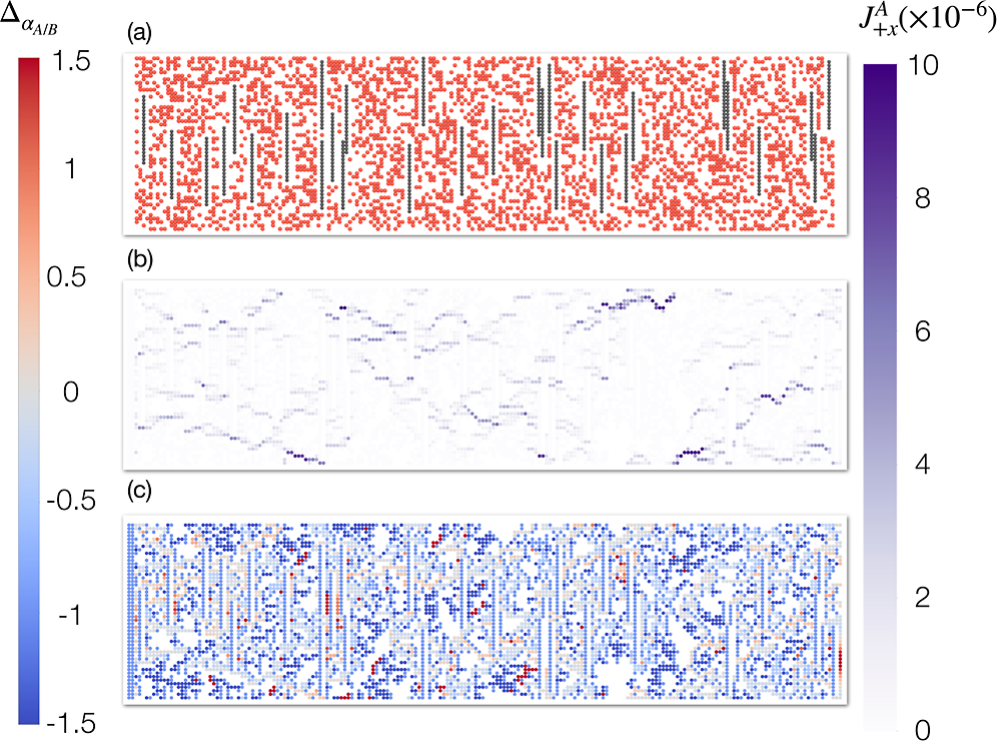
Snapshot of an MMM built from polymer membrane showed in Figure 5a. This MMM has ϕ_*f*_ = 6% and IG = 1 and the fillers are arranged vertically. The color bar shows the local selectivity difference and flux in the +*x* direction on the left and right sides, respectively.

Figure 6a shows a snapshot of a typical MMM with vertically arranged fillers based on the polymer membrane demonstrated in Figure 5a. This MMM has the exact same parameters as the MMM from Figure 5d (ϕ_*f*_ = 6.0% and IG = 1), and the two systems differ only in the arrangement of the fillers. From Figure 6b, the vertically arranged fillers open more passages in the original polymer, but since the fillers are nonpermeable, they also hinder the direct transport of the fluids, making it a much less permeable structure compared to the horizontally arranged fillers. Furthermore, unlike the MMM shown in Figure 5f where the perm-selectivity is dominated by the channels around the fillers, we do not observe strong selectivity effects in the vicinity of the vertically aligned fillers and the overall selectivity of the structure is similar to the original, pure polymer material.

**Figure 7:**
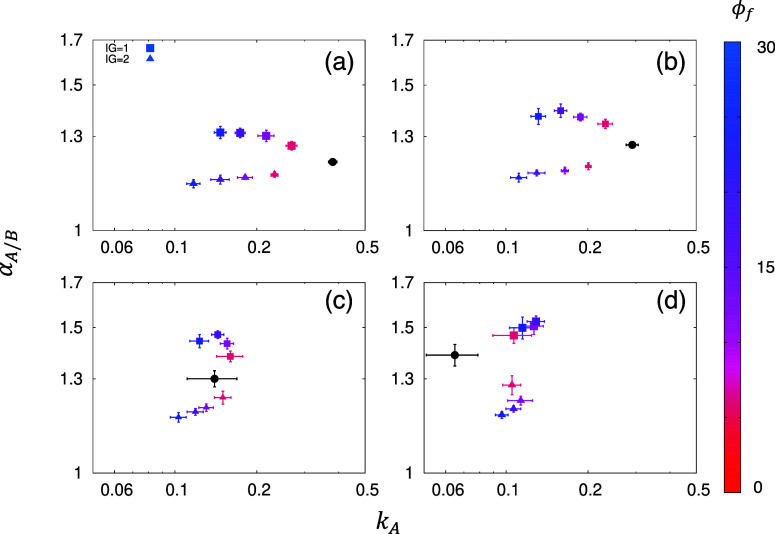
Perm-selectivity as a function of permeability for species *A*. Panel (a–d) shows results for MMMs with vertically arranged filler elements based on the reference systems with ϕ_*p*_ = 0.25, 0.30, 0.35, and 0.40, respectively. The black circles are the results for the unmodified, reference systems. The color bar shows the filler volume fraction ϕ_*f*_ (%). The error bars are the standard deviation.

Figure 7a–d shows the separation performance for these systems with the initial polymer membranes characterized by ϕ_*p*_ = 0.25, 0.30, 0.35, and 0.40, respectively. Here, polymers with *C*_4_ parameters are combined with the fillers. We observe that the insertion of the filler elements in the system with lower ϕ_*p*_ values, or in other words, a membrane with higher porosity, decreases the permeability compared to the reference system. This suggests that the presence of vertically aligned filler elements, even with the IGs, restricts the fluid flow within the membrane. However, for a polymer with initial ϕ_*p*_ = 0.40, where the fluid transport is already very constrained, introducing filler elements may result in a slightly positive impact on the permeability, depending on the value of the IGs. For IG = 1, the increase in the filler loading still creates additional passages for gas transport without losing the separation performance of the structure. Increasing the size of the IG leads to structures also featuring improved permeability; however, the weaker effective solid–fluid interactions due to the lower combined density of polymer and filler sites lead to diminished selectivity, which is mostly driven by the difference in solid–fluid interactions for different species. In addition, a direct comparison between the influences of horizontally and vertically arranged fillers can be made between Figures 4d and 7c where they feature the same base polymer membrane. As a result, all of the cases tested indicate that MMMs with horizontally arranged fillers have a better performance in both permeability and perm-selectivity. It is important to mention that the analysis of the experimental data for vertically aligned 2D nanoflakes (oriented perpendicular to the fluid flow) also suggests that the fluid transport through the defects (holes) in the filler elements plays an important role in the overall separation behavior.^34,36^ This mechanism based on transport within the filler elements and size selectivity is not considered in this work.

## Conclusions

In summary, in this paper, we take a close look at how the separation performance of MMMs improves with the addition of 2D nanoflakes as filler elements. Using DMFT, we extensively studied MMMs based on various model polymer structures, different filler loadings, varying extent of adhesion between them, and different orientations of the filler elements.

Our findings indicate that the degree of adhesion, as captured by IGs, significantly impacts the permeability of MMMs. When IGs are present between materials and with a reasonable filler loading, increasing the filler amount enhances permeability. This effect is clearly observed when visualizing the fluid flow patterns in MMMs. The filler elements regularize fluid flow patterns, creating directional transport passages with fast flow compared to the corrugated pathways in the original polymer. However, it is important to highlight that, in general, poor adhesion between the polymer host and the fillers can have detrimental effects on membrane performance. This poor adhesion may not only introduce large, macroscopic IGs but also lead to other structural defects, such as demixing of the polymer and fillers, filler precipitation, and impaired mechanical strength, among other issues. As a result, various methods have been proposed to address this challenge.^37,105^

In contrast, this study suggests that molecular-size interfacial channels can significantly enhance the separation of MMMs based on relatively inexpensive, nonporous additives. Achieving this, however, requires precise control of the dimensions and chemistry of the interfacial regions. As of now, engineering IGs remains an open scientific challenge.

Furthermore, the orientation of the nanoflakes profoundly affects the separation performance. Similar to experimental studies,^34^ we observed that fillers aligned parallel to the fluid flow significantly enhance permeability. In contrast, even with IGs present, vertical alignment tends to impede fluid transport, especially in polymers with a low initial porosity.

## Supporting Information Available

The Supporting Information provides a table summarizing the total solid fraction for different MMM systems (PDF)
