# Supplementary material for: How 2D Nanoflakes Improve Transport in Mixed Matrix Membranes: Insights from a Simple Lattice Model and Dynamic Mean Field Theory
Source: ACS Appl Mater Interfaces. 2024 Feb 3;16(6):8184–95. doi: 10.1021/acsami.4c00661 (PMC10875652; doi:10.1021/acsami.4c00661)
Supplement: Supplementary file 1 — am4c00661_si_001.pdf [file am4c00661_si_001.pdf]

Supporting Information: How 2D nanoflakes improve transport in mixed matrix membranes: insights from a simple lattice model and dynamic mean field theory

Tianmu Yuan* and Lev Sarkisov

Department of Chemical Engineering, Engineering Building A, The University of Manchester, Manchester, M13 9PL, UK

E-mail: tianmu.yuan@manchester.ac.uk

Total solid fraction

Table S1 shows the total solid fraction ϕ_s defined as $\phi_s = \phi'_p + \phi_f$ for the MMMs, after we have inserted the fillers with IGs. The term ϕ'_p is the polymer solid fraction after the insertion of the filler IGs. Each ϕ_s is averaged over 9 independent configurations.

Table S1: Total solid fraction (filler and polymer) ϕ_s in the MMMs with different polymer, IGs, and filler fraction.

ϕ_p, IG \ ϕ_f	6%	12%	18%	24%	30%
0.25, 0	0.2951	0.3401	0.3850	0.4302	0.4750
0.25, 1	0.2632	0.2863	0.3135	0.3414	0.3780
0.25, 2	0.2340	0.2373	0.2588	0.2925	0.3328
0.30, 0	0.3412	0.3849	0.4259	0.4685	0.5097
0.30, 1	0.3042	0.3171	0.3369	0.3597	0.3950
0.30, 2	0.2658	0.2596	0.2708	0.3026	0.3414
0.35, 0	0.3890	0.4288	0.4672	0.5069	0.5454
0.35, 1	0.3447	0.3461	0.3642	0.3802	0.4082
0.35, 2	0.3023	0.2834	0.2851	0.3101	0.3436
0.40, 0	0.4355	0.4721	0.5065	0.5436	0.5806
0.40, 1	0.3839	0.3824	0.3860	0.4022	0.4270
0.40, 2	0.3356	0.3092	0.3063	0.3217	0.3514
